# Long Non-coding RNAs in Prostate Cancer with Emphasis on Second Chromosome Locus Associated with Prostate-1 Expression

**DOI:** 10.3389/fonc.2017.00305

**Published:** 2017-12-12

**Authors:** Alessia Cimadamore, Silvia Gasparrini, Roberta Mazzucchelli, Andrea Doria, Liang Cheng, Antonio Lopez-Beltran, Matteo Santoni, Marina Scarpelli, Rodolfo Montironi

**Affiliations:** ^1^Section of Pathological Anatomy, School of Medicine, Polytechnic University of the Marche Region, United Hospitals, Ancona, Italy; ^2^Department of Pathology and Laboratory Medicine, Indiana University School of Medicine, Indianapolis, IN, United States; ^3^Department of Pathology and Surgery, Faculty of Medicine, Universidad de Córdoba, Córdoba, Spain; ^4^Oncology Unit, Macerata Hospital, Macerata, Italy

**Keywords:** second chromosome locus associated with prostate-1, metastatic prostate cancer, long non-coding RNA, lethal prostate cancer, prognostic biomarker, marker of aggressiveness

## Abstract

Long non-coding RNAs (lncRNAs) are a class of RNA with transcripts longer than 200 nucleotides that lack functional open reading frames. They play various roles in human carcinoma, such as dysregulating gene expression in prostate cancer (PCa), which results in cancer initiation, development, and progression. The non-coding RNA SChLAP1 (second chromosome locus associated with prostate-1) is highly expressed in approximately 25% of PCas with higher prevalence in metastatic compared to localized PCa. Its expression is detectable non-invasively in PCa patient urine samples. Experimental data suggest that targeting SChLAP1 may represent a novel therapeutic application in PCa. This contribution focuses on the role of lncRNAs SChLAP1 expression in PCa diagnosis and prognosis.

## Introduction

Prostate cancer (PCa) is the most commonly diagnosed cancer and the second major cause of cancer death in man (1). It is characterized by a wide and heterogeneous spectrum of clinical behaviors, ranging from indolent to aggressive forms. The clinical and morphological methods and features currently used in the routine show a low-predictive value concerning the definition of its level of aggressiveness (2, 3). Predictive and prognostic markers can be determined with clinical and pathological parameters, such as serum prostate-specific antigen (PSA), Gleason score (GS), and TNM stage. Due to PCa heterogeneity, patients with similar TNM stage, GS, and PSA could show opposite outcomes (4).

Additional predictive and prognostic markers are needed to distinguish high-risk from low-risk PCa patients. To this end, genetic and epigenetic investigations have been made to understand the complex genomic landscape of PCa in order to improve its diagnosis and prognosis and to define the potential role of new therapeutical targets (5). Long non-coding RNAs (lncRNAs), a class of RNA with transcripts longer than 200 nucleotides without functional open reading frames, play various roles in human carcinoma (6, 7).

This contribution focuses on the role of lncRNAs second chromosome locus associated with prostate-1 (SChLAP1) expression in PCa diagnosis and prognosis.

## Long Non-Coding RNAs

Long non-coding RNAs are a class of RNA with transcripts longer than 200 nucleotides that lack functional open reading frames (6). Based on their locations in the genome relative to protein-coding genes, lncRNAs have been subdivided into intergenic and intragenic. Intragenic lncRNAs can be further subclassified as exonic, intronic, and overlapping lncRNAs (8). Since protein-coding genes have been the focus of most research, the functional role of lncRNAs has been either underestimated or neglected (9).

As shown by Rinn and Chang, “More than 90% of human genome transcripts, including lncRNAs, do not code for proteins” (10). However, accumulating evidence suggests that lncRNAs play a role in the development of various types of cancers, such as PCa, hepatocellular carcinoma, non-small cell lung cancer, leukemia, colon carcinoma, and breast cancer (11–16).

While the mechanism of many lncRNAs remains to be elucidated, it has become clear that lncRNAs contribute to dysregulation of gene expression in PCa, thus resulting in cancer initiation, development, and progression (17).

## lncRNAs in Prostate Cancer

Elucidating the roles of lncRNAs in PCa holds great promise for early detection, prevention, and treatment. A well-known example is prostate cancer antigen3 (PCA3), also known as DD3, initially discovered *via* expression profiling of prostate sample (18). PCA3 has been extensively studied as a PCa-specific biomarker in body fluids. PCA3 urine RNA assay predicts biopsy status and histopathological characteristics (19). However, it does not predict outcomes such as recurrence and metastasis.

Another lncRNA investigated in PCa is metastasis-associated lung adenocarcinoma transcript-1 (MALAT-1), originally known to be overexpressed in patients at high risk for non-small cell lung cancer metastasis, as its name implies (20, 21). Its expression is found in many other human solid tumors having close correlation with invasiveness and metastasis (22–26). Ren and colleagues found that MALAT-1 is overexpressed in PCa compared to adjacent normal tissue (20). MALAT-1 expression is significantly higher in castration-resistant PCa (CRPCa) than in primary prostate tumor. Its expression increases from hormone sensitive to CRPCa. The same group of authors showed that this lncRNA could be a promising therapeutic target in patients with CRPCa. The intratumoral administration of therapeutic MALAT-1 siRNA suppressed CRPCa growth and metastasis *in vivo*, and prolonged the survival of tumor bearing mice (20).

It has also been shown that urine MALAT-1 is an independent predictor of PCa, more accurate than routine PSA. Its use would prevent one-third of unnecessary biopsies in PSA 4–10 ng/ml cohorts, without missing any high-grade PCa (27). Furthermore, in 192 plasma samples, MALAT-1 achieved high diagnostic accuracy in predicting prostate biopsy outcomes and, therefore, it might also be utilized as a plasma-based biomarker for PCa detection (28).

Recently, Zhao et al. investigated the expression profile of FALEC, another lncRNA, in PCa. Like other lncRNAs, its expression is significantly higher in PCa than adjacent normal parenchyma. Its downregulation inhibits cell proliferation, migration, and invasion (29).

Similar results were obtained with CCAT2, a lncRNA involved in proliferation, migration, and invasion of PCa cells. In particular, it was demonstrated that silencing of CCAT2 was able to inhibit N-cadherin, vimentin expression, and improve the expression level of E-cadherin, thus leading to the stimulation of epithelial-mesenchymal transition. High expression level of CCAT2 correlates with poor overall survival and progression-free survival and could be considered an independent prognostic factor in patients with PCa (30). Another promising lncRNA is LOC400891 which showed high expression in patients with an advanced PCa and a shorter biochemical recurrence-free survival time (31).

An interesting feature of lncRNAs is that many of them are not PCa specific. For example, we can observe overexpression of lncRNA-ATB in gastric cancer (32), hepatocellular carcinoma (33), osteosarcoma (34), and other tumors. Its tissue overexpression is directly proportional with the histological grade, high preoperative PSA level, pathological stage, high GS, lymph node metastasis, angiolymphatic invasion, and biochemical recurrence in PCa patients (35).

### lncRNA-Based Signature

Signatures, based on microarray lncRNA expression profiling, have been recently developed and widely used in prediction of a series of tumor characteristics and outcomes in various cancer type (36, 37). However, due to its low expression characteristics, a single lncRNA analysis might be associated with false-positive result. To overcome this problem, Huang and colleagues have developed a risk score based on lncRNA expression profile (38). They found four lncRNAs are significantly associated with BCR-free survival. Among the four lncRNAs, two (RP11-108P20.4 and RP11- 757G1.6) were positively associated with BCR-free survival, while the remaining two (RP11-347I19.8 and LINC01123) were negatively associated with BCR-free survival. They estimated a risk score for each patient and then divided patients into a high-risk group and a low-risk group by using the median risk score as the cutoff point. The four-lncRNA signature has been shown to be a powerful prognostic factor, independent of age, tumor and lymph node status, GS, margin status, and adjuvant postoperative radiotherapy (38) in (Table 1).

**Table 1:** Other lncRNAs associated with prostate cancer.

PCA3	Urine marker useful to predict biopsy status and histopathological characteristics (19)
MALAT-1	Its expression increases from hormone sensitive to CRPCa (20). Useful plasma biomarker for PCa detection (28)
FALEC	Its inhibition decreases cell proliferation, migration, and invasion (29)
CCAT2	Its high expression levels correlates with poor overall survival and progression-free survival (30)
LOC400891	Its high expression correlates with shorter BCR-free survival time (31)
ATB	Its high expression correlates with preoperative PSA levels, pathological stage, GS, lymph node metastasis, angiolymphatic invasion, and BCR (35)
RP11-108P20.4	Positively associated with BCR-free survival
RP11-757G1.6	Part of the Four-lncRNA signature (38)
RP11-347I19.8	Negatively associated with BCR-free survival
LINC01123	Part of the Four-lncRNA signature (38)

*CRPCa, castration resistant; PCa, prostate cancer; BCR, biochemical recurrence; PSA, prostate-specific antigen; GS, Gleason score; lncRNAs, long non-coding RNAs*.

## Second Chromosome Locus Associated with Prostate-1

About 1,800 lncRNAs were identified by Presner et al. through the application of RNA sequencing techniques (i.e., transcriptome sequencing) on a consistent number of tissue samples. Of these 1,800 lncRNAs, 121 resulted transcriptionally dysregulated in PCa (39). Such 121 Prostate Cancer-Associated Transcripts represent an unbiased list of potentially functional lncRNAs associated with PCa. By performing a cancer outlier profile analysis to identify intergenic lncRNAs selectively upregulated in a subset of cancers, they found two lncRNAs, PCAT-109 and PCAT-114, both located on Chromosome 2q31.3 in a “gene desert,” a region of the genome that are lacking of protein-coding genes. Both genes showed “outlier profiles and ranked among the best outliers in PCa” (40). In particular, PCAT-114 was found to be overexpressed in prostate cell lines. It was named SChLAP1 after its genomic location (40). *Schlap1* gene has a transcript length of 24,484 Kb. The complete gene is composed of 7 exons and 1,675 nucleotides. The primary transcript (isoform 1) is composed of 5 exons with a length of 1,436 nucleotides. As a result of a spicing process, a total of 8 isoforms were found, with isoform 1, isoform 2, and 3 accounting for >90% of transcripts (41). RNA-seq, performed on 27 different tissue samples from 95 human individuals, showed that SChLAP1 expression was highly specific for prostate tissue, being present at minor levels in bladder, kidney, and testis samples (42).

### SChLAP1’s Working Mechanisms: Interaction with SWI/SNF Complex and miR-198

*In vitro* and *in vivo* gain-of-function and loss-of-function experiments have shown that SChLAP1 plays a crucial role in cancer cell invasiveness and metastasis, antagonizing the activity of the SWI/SNF chromatin-modifying complex, a multiprotein system able to move nucleosomes at gene promoters. In particular, such experiments showed that the inactivation of SWI/SNF complex promoted cancer progression and that multiple SWI/SNF components were somatically inactivated in cancer (41, 43). As shown by Prensner, even though “other lncRNAs, such as HOTAIR and HOTTIP, are known to assist epigenetic complexes such as PRC2 and MLL by facilitating their genomic binding and enhancing their functions, SChLAP1 is the first lncRNA that impairs a major epigenetic complex with well-documented tumor suppressor function” (41, 44).

*In vivo*, SChLAP1 has been shown to be implicated in tumor cell proliferation and metastasization, as evidenced by both the reduction of tumor growth kinetics and the decreased number and dimensions of metastatic sites as a consequence of the intracardiac injection of 22Rv1 cells with SChLAP1 knockdown in CB-17 SCID mice (41).

**Figure 1:**
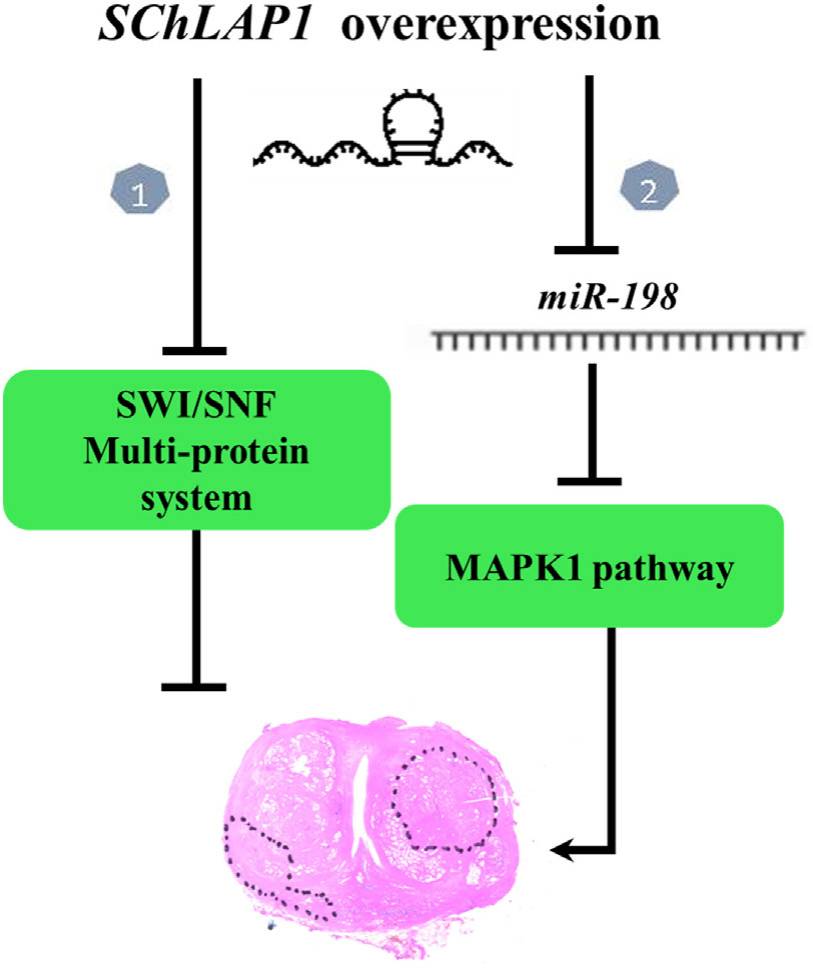
Working model of second chromosome locus associated with prostate-1 (SChLAP1) in prostate cancer (PCa). There are two pathways. In Pathway 1, loss of SWI/SNF functionality promotes cancer progression (41). In Pathway 2, miR-198 might exert its anticancer effect through inhibition of MAPKs signaling pathway. SChLAP1 acts as a negative regulator in the expression of miR-198 and subsequently modulates the MAPK1 signaling pathway in PCa (49).

Recent studies have shown interaction between SChLAP1 and miR-198. MiR-198 is downregulated in many cancers, such as gastric cancer, lung cancer, and hepatocellular carcinoma (45–47). miR-198 suppress the proliferation and invasion of colorectal carcinoma (48). miR-198 might exert its anticancer effect through inhibition of MAPKs signaling pathway (49). In PCa tissue, a low expression of miR-198 was found. As shown by Li et al., “knockdown of SChLAP1 significantly increased the expression of miR-198 and SChLAP1 overexpression markedly decreased it. Thus, SChLAP1 acted as a negative regulator in the expression of miR-198” and subsequently modulated the MAPK1 signaling pathway in PCa (49) (Figure 1). Transfecting PCa cells with a designed-specific siRNA to knockdown SChLAP1 expression, investigators have obtained, as expected, a significantly reduction in cell proliferation together with an increase in apoptosis-associated proteins. Furthermore, SChLAP1 knockdown determined a decrease of MMP-9, MMP-14, and VEGF expressions both *in vitro* and *in vivo*, confirming its involvement in cancer invasiveness and metastasis (49). All such findings show multiple interactions between SChLAP1 and factors involved in oncogenesis and cancer progression and explain the mechanism through which SChLAP1 promotes migration and invasion of PCa (50). Understanding this molecular pathway is essential for exploring new potential strategies for early diagnosis and therapy.

### SChLAP1: Predictor of Aggressive PCa

Second chromosome locus associated with prostate-1 is highly expressed in approximately 25% of PCa, expression being higher in metastatic compared to localized prostate cancers. It was associated with ETS gene fusions (41). Multivariate and univariate regression analyses have demonstrated that SChLAP1 expression is an independent predictor of PCa aggressiveness with highly significant hazard ratios for predicting BCR, clinical progression to systemic disease, and PCa-specific mortality, compared to other clinical factors such as advanced clinical stage and the GS (41). Moreover, SChLAP1 expression was significantly associated with extracapsular extension, seminal vesicle invasion, and positive surgical margin status (40).

Validation in three-independent cohorts has confirmed the prognostic value of SChLAP1 for metastasis. On multivariate modeling, SChLAP1 expression independently predicted metastasis within 10 years, death within 10 years, and biochemical recurrence within 5 years with odds ratios, comparable to GS. Among all known genes, the lncRNA SChLAP1 ranked first for elevated expression in patients with metastatic progression by receiver-operator-curve area-under-the-curve analyses and was the only prostate-specific gene, ideal for development as a non-invasive biomarker (40).

Similar results have been obtained by Mehra and colleagues using a novel RNA *in situ* hybridization (ISH) assay for detection of SChLAP1 in formalin-fixed, paraffin-embedded tissue (51). They found that high SChLAP1 expression independently predicts biochemical PCa recurrence after radical prostatectomy in patients with clinically localized PCa and that it is associated with the development of lethal PCa.

Interestingly, high SChLAP1 expression is associated with lethal PCa among patients with non-advanced clinical tumor stage. Similarly, high SChLAP1 expression is associated with lethal PCa among patients with low grade tumors (GS ≤7). However, using SChLAP1 as prognostic test, investigators have obtained a sensitivity around 24% and a specificity of 94% in the group of non-advanced clinical tumor stage and in the group with a 6–7 GS (52). Considering the low sensitivity of the test in the identification of an aggressive disease in contrast with low-risk morphological features, the evaluation of SChLAP1expression alone does not seem to improve treatment decision. In conjunction with other prognostic tools, SChLAP1 has been shown to improve upon established clinical algorithms for the risk stratification of PCa patients, specifically the CAPRA-S score (53, 54), i.e., one of the best clinic-pathological models to date. SChLAP1 further improves prediction upon both the Decipher test 50 and the CCP gene signature (55, 56).

Recently, Chua et al. have investigated SChLAP1 expression in subsets of PCa characterized by cribriform architecture (CA) and intraductal carcinoma (IDC), features both associated with increased risks of biochemical relapse and metastasis. Besides the histological presentation, IDC/CA+ cancers harbor an increased percentage of genome aberration (PGA). This is in agreement with the observation that tumors with IDC or genomic instability have a greater metastatic potential. Using mRNA abundance analyses and assessing >25,000 genes, they found that SChLAP1 was surprisingly the only gene with more than threefold higher expression in IDC/CA+ compared to IDC/CA− cancers (57).

The association of SChLAP1 expression within IDC/CA+ tumors has also been further demonstrated by SChLAP1 RNA-ISH in prostatectomy TMA-cores. The SChLAP1+, IDC/CA+ subgroup has shown a significant increase of biochemical relapse, independent of PGA. Combining histology features of cribriform architecture and intraductal carcinoma with genomic instability or SChLAP1 expression can stratify patients for recurrence more accurately than any parameter alone. Interestingly, SChLAP1 RNA-ISH diffuse expression has been observed in the cribriform architecture and intraductal carcinoma and in the adjacent invasive adenocarcinoma. This further supports a field defect and a common clonal ancestor to both histopathologies (58).

### SChLAP1 in Urine Sediments

Second chromosome locus associated with prostate-1 expression is detectable non-invasively in PCa patient in urine samples. Its expression is both more frequent and more highly elevated in GS 7 compared to GS 6 patients even if it is less sensitive than PCA3 and TMPRSS-ERG gene fusion (40). “SChLAP1 expression may complement existing urine diagnostic assays, including PCA3 and TMPRSS2-ERG, and that clinical application of a SChLAP1 urine test would be most effective in conjunction with these, and potentially other, urine assays” (59).

### SChLAP1 As a Potential Drug Target

RNA interference (RNAi) technology using short interfering RNA (si-RNA) has shown great potential in the treatment of cancers through silencing of specific genes. *In vitro* and *in vivo* experiments have demonstrated that SChLAP1-knockdown promoted apoptosis and inhibited cell proliferation and invasion (41, 49). SChLAP1 is also overexpressed in bladder cancer compared to paired normal bladder tissues. Cell transfected with SChLAP1 siRNA showed growth arrest, apoptosis, and migration inhibition, suggesting oncogenic roles in bladder cancer and a potential therapeutic target (Table 2) (60). Such results might be the starting point to investigate the therapeutical potential of antagonizing SChLAP1 oncogenic functions (60).

**Table 2:** SChLAP1 expression in prostate cancer.

Reference	Result	Method	No. specimens
Prensner (39)	121 novel lncRNA loci (out of >1,800) were aberrantly expressed in PCa tissues Only two, PCAT-109 and PCAT-114, showed striking outlier profiles and ranked among the best outliers in PCa	RNA-Seq; COPA	102 PCa tissue samples and cell lines

Prensner (41)	SChLAP1 expression is an independent predictor of PCa aggressiveness with highly significant hazard ratios for predicting BCR, CP, and PCSM SChLAP1 antagonizes tumor-suppressive functions of the SWI/SNF complex	qPCR	235 RP localized PCa

Prensner (40)	SChLAP1 expression independently predicted metastasis, PCa-specific death, and BCR with OR comparable to GS SChLAP1 expression was detectable non-invasively in urine samples and associated with higher-risk patients	RNA extraction, microarray hybridization; qPCR	1,008 patients. Three independent cohorts; 230 urine sediment samples

Mehra (51)	SChLAP1 expression is enriched in samples from tumors with high GSs (≥8) compared to tumors with lower GSs High SChLAP1 expression independently predicts BCR (PSA relapse) after RP	ISH	160 clinically localized PCa

Mehra (52)	High SChLAP1 expression is significantly associated with a higher risk of lethal PCa and PCa-specific death independent of age at diagnosis, GS, and pathologic stage High SChLAP1 expression is associated with lethal PCa among patients with non-advanced clinical tumor stage, but not among patients with advanced clinical tumor stage	ISH	937 PCa patients

Zhang (60)	SChLAP1 was overexpressed in bladder cancer tissues compared to paired normal bladder tissues Cell growth arrest, apoptosis induction, and migration inhibition were also observed in bladder cancer cells after transfection with SChLAP1 siRNA	qPCR; CCK-8 assay, flow cytometry analysis, and wound healing assay	Bladder cancer T24 and 5,637 cells

Chua (57)	SChLAP1 was the only gene expressed at >3-fold higher in intraductal carcinoma (IDC) and cribriform architecture (CA) PCa than in IDC/CA - tumors	Profiling of mRNA abundance, ISH	1,325 localized PCa

Li (49)	Knockdown of SChLAP1 promoted apoptosis and inhibited cell proliferation and invasion *in vitro* and *in vivo* SChLAP1 acted as a negative regulator in the expression of miR-198 and accelerates the proliferation and metastasis of PCa promoting the MAPK1 pathway	Not available	Not available

*RNA-Seq, next generation transcriptome sequencing; COPA, cancer outlier profile analysis; PCa, prostate cancer; qPCR, quantitative Polymerase Chain Reaction; FFPE, formalin-fixed paraffin embedded; BCR, biochemical recurrence; CP, clinical progression; PCSM, prostate cancer-specific mortality; GS, Gleason score; OR, odds ratio; RP, radical prostatectomy; ISH, in situ hybridization; SChLAP1, second chromosome locus associated with prostate-1; lncRNAs, long non-coding RNAs*.

## lncRNA in Diagnosis, Prognosis, and Treatment: Pros and Cons

There are *Pros* and *Cons* with the use of lncRNA in the diagnosis and treatment of PCa patients. Indeed, lncRNA may represent a useful biomarker that can give to clinicians fundamental information on tumor biological behavior and aggressiveness, leading to the possibility of designing personalized and tailored strategies for a single PCa patient. This may also allow an optimization of patients’ outcome and to avoid useful costs and consequences of not effective therapies for PCa patients.

As far as the *Cons*, tumor aggressiveness is the result of a complex process that involves lncRNA and a variety of driver genes, leading to the necessity for uropathologists to test and validate not a single driver gene, but a panel of genes with direct consequences on the relative costs of these procedures.

## Conclusion

In conclusion, research on lncRNAs in PCa is at its onset. However, as shown in this review:

- The first set of data has revealed central roles with clinical significance for lncRNAs in different stages of the disease.
- There is evidence that lncRNAs, including SChLAP1, are critical in PCa development and progression.
- Concerning future perspective, mainly based on experimental data, targeting SChLAP1 may become a novel therapeutic application in PCa.

## Author Contributions

RM: conception and design. AC and SG: drafting the manuscript. AD and RMa: acquisition of data. LC and AL-B: critical revision of the manuscript. MS: supervision.

## Conflict of Interest Statement

The authors have no relevant affiliations or financial involvement with any organization or entity with a financial interest in or financial conflict with the subject matter or materials discussed in the manuscript. This includes employment, consultancies, honoraria, stock ownership or options, expert testimony, grants or patents received or pending, or royalties. No writing assistance was utilized in the production of this manuscript.
